# Camera Stabilization in 360° Videos and Its Impact on Cyber Sickness, Environmental Perceptions, and Psychophysiological Responses to a Simulated Nature Walk: A Single-Blinded Randomized Trial

**DOI:** 10.3389/fpsyg.2019.02436

**Published:** 2019-11-01

**Authors:** Sigbjørn Litleskare, Giovanna Calogiuri

**Affiliations:** Faculty of Social and Health Sciences, Inland Norway University of Applied Sciences, Elverum, Norway

**Keywords:** green exercise, virtual reality, restorative environments, environmental perception, immersive virtual environments

## Abstract

Immersive virtual environments (IVEs) technology has emerged as a valuable tool to environmental psychology research in general, and specifically to studies of human–nature interactions. However, virtual reality is known to induce cyber sickness, which limits its application and highlights the need for scientific strategies to optimize virtual experiences. In this study, we assessed the impact of improved camera stability on cyber sickness, presence, and psychophysiological responses to a simulated nature walk. In a single-blinded trial, 50 participants were assigned to watch, using a head-mounted display, one of two 10-min 360° videos showing a first-person nature walk: one video contained small-magnitude scene oscillations associated with cameraman locomotion, while in the other video, the oscillations were drastically reduced thanks to an electric stabilizer and a dolly. Measurements of cyber sickness (in terms of both occurrence and severity of symptoms), perceptions of the IVE (presence and perceived environmental restorativeness), and indicators of psychophysiological responses [affect, enjoyment, and heart rate (HR)] were collected before and/or after the exposure. Compared to the low-stability (LS) condition, in the high-stability (HS) condition, participants reported lower severity of cyber sickness symptoms. The delta values for pre–post changes in affect for the LS video revealed a deterioration of participants’ affect profile with a significant increase in ratings of negative affect and fatigue, and decrease in ratings of positive affect. In contrast, there were no pre–post changes in affect for the HS video. No differences were found between the HS and LS conditions with respect to presence, perceived environmental restorativeness, enjoyment, and HR. Cyber sickness was significantly correlated with all components of affect and enjoyment, but not with presence, perceived environmental restorativeness, or HR. These findings demonstrate that improved camera stability in 360° videos is crucial to reduce cyber sickness symptoms and negative affective responses in IVE users. The lack of associations between improved stability and presence, perceived environmental restorativeness, and HR suggests that other aspects of IVE technology must be taken into account in order to improve virtual experiences of nature.

## Introduction

Nature is believed to have intrinsic qualities that promote health and well-being (Bowler et al., 2010; Bosch and Bird, 2018), which has led to increased interest in nature exposure as a research area. In the past two decades, interest and concerns have been raised regarding the possibility of using *virtual nature* to supplement exposure to real nature. Virtual nature might be a tool to increase the exposure to restorative nature experiences in an increasingly urbanized population and might even contribute to the reconnection of people to real nature, as studies show that acute bouts of green exercise (i.e., any physical activity performed while being exposed to nature) can increase people’s feelings of nature connectedness (Mayer et al., 2009) and future intention to exercise and/or visit natural environments (Hug et al., 2008; Calogiuri and Chroni, 2014; Calogiuri et al., 2015). The effectiveness of this technology as an instrument in palliative care has been documented (Chirico et al., 2016; White et al., 2018). This technology could also address some of the major methodological issues inherent to environmental research (Smith, 2015), particularly in studies of natural environments (Lahart et al., 2019), as research related to outdoor environments does not allow strict control of confounding factors such as weather conditions, temperature, ambient noises, etc. To date, however, it remains unclear to what extent virtual nature can provide benefits equivalent to those provided by experiences in real nature, while on the other hand, concerns have been raised regarding the possible undesired effect of further distancing people from real nature (Levi and Kocher, 1999). Moreover, in spite of the fact that virtual reality has become a phenomenon of mass consumption, a series of side effects that can undermine the quality of users’ experience yet remain to be addressed. These side effects are also known to mask potential effects in studies of virtual environments, thus limiting the usefulness of virtual technology in environmental research.

### The Effectiveness of Virtual Nature

The potential of and the interest in virtual nature have been accelerated by the upcoming and continuous development of immersive virtual environments (IVEs) technology. IVEs are virtual environments that “surround an individual and create the perception that they are enclosed within and interacting with environments that provide a continuous stream of stimuli” (Smith, 2015). Head-mounted displays, devices with a motion sensor that allows a 360° vision of a virtual world while eliminating the visual contact with external reality, are central elements of IVE technology. These displays are considered more immersive than traditional displays and are believed to increase *presence*, i.e., the illusion of “being there” (Weech et al., 2019), which is considered a key element of the effectiveness of virtual environments (Steuer, 1992). The popularity of IVEs and head-mounted displays follows the introduction of affordable technology that not only provides the opportunity to immerse oneself in pre-set IVEs, but also allows the creation of new IVEs using special 360°cameras and freely available and customizable applications. *Static* IVEs (i.e., IVEs in which the perspective of the viewer is stationary, but still allow a 360° range of vision) have been shown to be able to replicate some of the positive psychological effects of exposure to real nature (Chirico and Gaggioli, 2019; Yu et al., 2018), such as helping people reduce stress (Liszio and Masuch, 2018) or experiences of pain (Chirico et al., 2016; White et al., 2018). The effectiveness of virtual environments has further been confirmed in studies of urban environments, in which virtual images and soundscapes are perceived as comparable to the real environment (Maffei et al., 2016; Hong et al., 2019).

While the literature provides increasingly strong support for the effectiveness of static natural IVEs, more challenges are encountered in relation to *dynamic* IVEs (i.e., IVEs in which the perspective of the viewer moves while allowing a 360° range of vision, for example, a simulated walk in a natural environment). Previous studies have found some psychological benefits of exposure to dynamic IVEs administered either in sedentary conditions (Valtchanov et al., 2010) or in combination with physical movement (Plante et al., 2003, 2006). The head-mounted displays used in these studies, however, only allowed a 60° to 65° range of vision, thus not fully engaging the viewers’ peripheral vision. To the best of our knowledge, only one study has used *dynamic* 360° IVEs to compare a simulated nature walk (both in combination and not in combination with actual physical movement) to an actual outdoor walk in the same environment (Calogiuri et al., 2018). The results from this study suggest that the technology might have the potential to elicit psychological responses similar to those experienced in real nature, as in fact the participants assigned equivalent levels of perceived environmental restorativeness in the IVE as in the real environment and spontaneously walked at the same pace in both conditions. However, the users reported a significant deterioration of their psychological state after exposure to the IVE, with a decrease in positive emotions (tranquility and positive affect) and an increase in negative emotions (fatigue and negative affect). Such dramatic psychological responses appeared to be primarily associated with the experience of a phenomenon known as *cyber sickness*.

### The Impact of Cyber Sickness on the Effectiveness of Virtual Nature

Cyber sickness mimics the symptoms of motion sickness, inducing feelings of dizziness, nausea, and general discomfort (Smith, 2015), and can be viewed as a specific type of visually induced motion sickness (Kennedy et al., 2010). There has been an increase in reported cases of cyber sickness in recent years, which may relate to the fact that more technologically advanced displays, such as head-mounted displays, generally induce higher levels of cyber sickness (LaViola, 2000; Sharples et al., 2008). There are two prevailing theories regarding the cause of cyber sickness. The *sensory conflict theory* postulates that cyber sickness is caused by sensory conflict between visual, vestibular, and proprioceptive inputs (Reason and Brand, 1975). Another explanation to how cyber sickness may originate is provided by the *postural instability theory*, which states that long periods without postural control will cause cyber sickness (LaViola, 2000). The negative effects caused by cyber sickness is a concern in studies of virtual environments as it is likely to act as a competing factor to the potential benefits of exposure to virtual nature. For example, recent research suggests that cyber sickness is inversely related to presence and that reducing cyber sickness might improve presence (Weech et al., 2019). Cyber sickness may also compete with the environmental perceptions. According to Rachel and Stephen Kaplan’s Attention-Restoration Theory, environments characterized by qualities of fascination, compatibility, extent, and feeling of being away have the potential to elicit cognitive restoration and positive affective responses (Kaplan, 1989, 1995). However, Calogiuri et al. (2018) compared affective responses after exposure to real and virtual natural environments and found that the real environment induced a more positive affective response compared to the virtual environment, despite the fact that the participants perceived the virtual environment as having restorative properties equivalent to those perceived in the real environment. Calogiuri et al. (2018) further demonstrated medium to high correlations between cyber sickness and participants’ affective responses, which suggest that cyber sickness disrupted any potential positive effects related to the restorative value of the virtual environment. The discomfort caused by cyber sickness and its potential impact on affect and presence limit the application of VR in health promotion and research purposes, and highlight the need for scientific strategies to optimize virtual experiences. Interestingly, recent advances in handheld stabilizing devices specifically designed for 360° cameras provide a potential strategy. These stabilizing devices reduce camera oscillations, which might reduce levels of cyber sickness.

### The Impact of Scene Oscillations on Cyber Sickness

It has been hypothesized that the presence of scene oscillations might play a central role in the generation of cyber sickness during exposure to dynamic IVEs. Oscillations, which include movements of the scene displayed in the head-mounted displays on the horizontal and/or vertical axis (also known as “pitch and yaw”), are an issue in the development of dynamic IVEs, especially those developed using 360° cameras. Such oscillations may be generated as a consequence of poor stabilization of the camera (e.g., general vibrations) as well as vertical movements associated with the locomotion of the camera operator. Previous provocation studies, i.e., participants were exposed to a stimulus that was expected to provoke a negative response, have investigated the effect of oscillations using head-mounted displays (Lo and So, 2001; Bonato et al., 2009). In these studies, they intentionally created computer-generated IVEs with severe levels of oscillations and found that an increase in oscillations increases levels of cyber sickness (Lo and So, 2001; Bonato et al., 2009). It is not clear how the presence of oscillations leads to increased levels of cyber sickness. However, it has been suggested that when the viewer experiences the scene moving in one direction, it causes a feeling of self-motion (i.e., vection) in the opposite direction (Lo and So, 2001). This sense of self-motion perceived by the visual sensory system might cause sensory conflict with input from the vestibular and proprioceptive systems, as these sensory systems would not perceive any motion in this situation, thus linking the sickness-inducing effect of oscillations to the sensory conflict theory. Although this is just a theory and researchers debate whether or not vection actually is related to cyber sickness (Keshavarz et al., 2015), the studies of Lo and So (2001), and Bonato (2009) have established that high levels of artificially generated oscillations have a severe impact on cyber sickness. However, it is still unknown whether this applies to more practical situations and, in particular, to what extent minimizing oscillations can reduce levels of cyber sickness in 360° videos that are not intentionally designed to provoke a negative response.

### The Present Study

The purpose of the present study was to assess the extent to which improved camera stability, in dynamic 360° videos simulating a nature walk, could reduce cyber sickness and improve participants’ sense of presence and psychophysiological responses.

Our primary hypothesis was stated as follows:

A 360° video simulating a nature walk with a high level of camera stability will be associated with less severe symptoms of cyber sickness compared to a less stable video.

Our secondary hypotheses were stated as follows:

1.1. If a highly stable 360° video simulating a nature walk induces less cyber sickness, it will also be associated with higher levels of presence compared to a less stable video.
1.2. If a highly stable 360° video simulating a nature walk induces less cyber sickness, it will also be associated with a more positive psychophysiological response compared to a less stable video.

In addition, in relation to each hypothesis, we performed an exploratory correlation analysis investigating possible associations between the variables in order to evaluate possible pathways that can help explain the complex relation linking cyber sickness, environmental perceptions, and psychophysiological responses.

## Materials and Methods

This paper is structured in line with the CONSORT guidelines (Schulz et al., 2010).

### Participants

Estimation of required sample size was based on total score on the simulator sickness questionnaire (SSQ) from a preliminary pilot study done in our laboratory. The smallest meaningful between-group difference in total SSQ score was set to 20, with a pooled standard deviation of 30. α-level and desired power was set to 0.05 and 80%, respectively. Based on this calculation, an approximate sample size of 50 was deemed appropriate. Participants were recruited by the first author among students and non-scientific employees at the Inland Norway University of Applied Sciences (Elverum), but also a few participants outside the University during the months of June and August–October. Inclusion criteria were as follows: being >18 years old, having normal or corrected-to-normal vision, having limited experience with VR (less than monthly use), and not having previous diagnosis of balance impairments. The final sample comprised 22 males and 28 females. Participants’ background characteristics are presented in Table 1. All participants signed a written informed consent prior to the experiment. The study was approved by the Norwegian Center for research data (reference number: 60451) and conducted according to the declaration of Helsinki.

**TABLE 1:** Baseline characteristics of the participants (*n* = 50).

	**LS (*n* = 25)**	**HS (*n* = 25)**

Male (*n*)	11	11
Female (*n*)	14	14

**Variables**	**M (SD)**	**M (SD)**
Age (years)	30.6 (11.6)	30.0 (11.3)
BMI (kg/m^2^)	25.8 (3.5)	24.6 (3.1)
Weekly physical activity (METs)	54.8 (22.3)	59.6 (26.4)

*LS = low stability video. HS = high stability video. BMI = body mass index.*

### IVE Technology and Experimental Conditions

The IVEs used for the experiment consisted of two 10-min-long 360° videos showing a first-person walk along a path in a natural environment. The playback was made using Samsung S7, with Android 7.0, mounted on a Samsung Gear head-mounted display (Samsung Gear VR SM-R323). The environment contained a fairly straight paved trial in natural surroundings alongside a river in Elverum. In addition to natural elements such as trees, grass, and water, the environment also included some buildings and a football field. An actual walk of the same duration along the same path has previously been reported to improve participant’s affect state, with significant reductions in fatigue and negative affect, but no changes in tranquility and positive affect (Calogiuri et al., 2018).

The two videos showed the same nature walk and differed only in the extent to which they contained frequent scene oscillations of small magnitude. Both IVEs were recorded in the same location using the same 360° video camera (Samsung gear 360 smc200, 3840 × 1920 resolution at 30 frames per second), in pleasant weather conditions. Measures were taken to ensure that all elements except camera oscillations would be as similar as possible. This includes recording the scene segments at the same time of year with similar levels of “greenness,” matching the lighting postproduction based on the subjective evaluation of the authors, matching start and end position of the videos, etc. The low-stability (LS) video was created using segments from a video used in a previous experiment (Calogiuri et al., 2018). To develop that video, a 360° camera was mounted on a modified Yelangu s60t mechanical handheld stabilizer and the video recorded was run through two software stabilizing programs as described in Calogiuri et al. (2018). This procedure was developed to minimize camera oscillations, but due to the oscillations associated with the camera operator locomotion as well as the mechanical nature of the stabilizer, oscillations of small magnitude still occurred along both the vertical and horizontal axis. In contrast, the high-stability (HS) video was recorded by mounting the 360° camera on an electronic gimbal handheld stabilizer (Feiyu Tech G360), which provided a high degree of stability, especially in the horizontal axis (pitch). The camera operator was also pushed along the path on a dolly to further minimize oscillations in the vertical axis (yaw). This procedure effectively eliminated pitch oscillations though, in order to align the camera positioning with the path, yaw oscillations of small magnitude still occurred sporadically (six times during the entire duration of the video). The two videos were similar in all aspects, but differed in both frequency and magnitude of oscillations.

### Instruments

#### Cyber Sickness

The occurrence of cyber sickness was measured with a dichotomous question: “are you cyber sick,” followed by a brief verbal explanation of the term, to which the participant responded either “yes” or “no” (Merhi et al., 2007; Munafo et al., 2017). The SSQ was used to measure the severity of the symptoms related to cyber sickness (Kennedy et al., 1993). As the name implies, the SSQ was designed to measure simulator sickness, but the questionnaire has seen widespread use in studies of cyber sickness as well (e.g., Draper et al., 2001; Merhi et al., 2007; Munafo et al., 2017). Participants were asked to rank, on a four-point Likert scale, the severity of 16 different symptoms such as “Headache” and “Increased salivation.” Scoring and analysis of the SSQ data were performed according to the recommendations of Kennedy et al. (1993). The scale showed adequate internal consistency for the total score (α = 0.94).

#### Presence and Environmental Perceptions

To assess participants’ sense of presence in the IVEs, we used a scale based on the approach of Nichols et al. (2000), which includes eight items related to presence in virtual environments. The items were formulated as statements as shown in Table 2, and participants were asked to rate their level of agreement to each statement on an 11-point Likert scale. Alongside the measure of presence, the extent to which the participants perceived the IVEs as having a potential for environmental restorativeness was assessed by administering the perceived restorativeness scale (Hartig et al., 2003). In the context of our study, this measure provided an indicator of the potential of the IVEs to elicit cognitive restoration. The scale consists of 16 items, rated on a 11-point Likert scale (0 = absolutely disagree, 10 = absolutely agree), which assess the subjective perception of four environmental qualities in line with Rachel and Stephen Kaplan’s Attention-restoration theory (Kaplan, 1989, 1995): Being away, which refers to the extent to which the environment provides a feeling of “being away” from everyday demands and concerns (two items, e.g., “It was an escape experience”); Fascination, the environment’s ability to capture the viewers’ effortless attention (five items, e.g., “The setting has fascinating qualities”); Coherence, the extent to which the elements in an environment combines to a coherent whole (four items, e.g., “There is too much going on”); and Compatibility, which describes how well a place fits people’s inclinations and interests (five items, e.g., “have a sense that I belong there”). The caption on top of the items explicitly stated “The following questions relate to the virtual environment.” The scale showed adequate internal consistency (α = 0.74–0.86).

**TABLE 2:** Items used to assess presence.

**Short name**	**Item**
Being there	In the computer-generated world, I had the sense of “being there”
Realism	I thought of the virtual environment as equal to the real environment
Sense of reality	The virtual world became more real or present to me compared to the real world. NB: by “real world,” we mean the room where you were undergoing the test
Awareness	During the “virtual walk,” I often thought of the other person(s) in the room with me
Other persons	It would have been more enjoyable to engage with the “virtual world” with no one else in the room
External noises	While I was doing the “virtual walk,” I paid much attention to other noises around me in the room
Flatness	The virtual world appeared flat and missing in depth
Movements lag	The lag, delay or difference between my movements and the movements in the “virtual walk” were disturbing

#### Psychophysiological Responses

The participants’ psychophysiological responses to the different IVEs were evaluated in relation to enjoyment, pre-to-post changes of affect, and heart rate (HR). Enjoyment was assessed after exposure to the IVE using a single-item inquiring: “on a scale from 1 to 10, how enjoyable was the activity you engaged in?” This measure has been used in studies investigating participants’ affective responses to green exercise, showing high correlation with measurements of perceived environmental restorativeness (Calogiuri et al., 2015, 2018). Participants’ affect was assessed before (Pre) and after (Post) exposure to the IVE using the Physical activity affect scale (Lox et al., 2000). The scale consists of 12 items (e.g., “energetic,” “calm,” “miserable,” and “tired”) that, in line with Russel’s circumplex model (Russell, 1980), are grouped in four components: Positive affect (positive valence, high activation), Tranquility (positive valence, low activation), Negative affect (negative valence, high activation), and Fatigue (negative valence, low activation). The scale showed, in general, adequate internal consistency for the different subscales (α = 0.68–0.87), though somewhat poor levels of internal consistency were found for Positive affect in the Pre assessment (α = 0.56) and Negative affect in both assessments (α = 0.56–0.56, in Pre and Post, respectively). Lastly, HR was recorded continuously over a 6-min period during exposure to the IVEs using a HR-monitor (Polar FT60M BLK WD) and extracted as beats per minute as a physiological indicator of stress (Allen et al., 2014; Duzmanska et al., 2018). It was decided to exclude the first and last couple of minutes of video exposure from the HR measurements to allow postural adaptation of HR in the beginning of the video, as the participants moved from a standing to a seated position, and due to concerns that the fact that the examiner reentered the experimental room at the end of the video would influence HR. Mean HR (HRmean), the mean of all individual measurements, and maximal HR (HRmax), the single highest value recorded, were automatically recorded by the HR monitor and used for further analyses.

#### Participants’ Background Characteristics

This information was collected in order to establish a general indication of the health and fitness status of the participants, and included sex, age, body mass index (BMI), and physical activity habits. Sex and age were self-reported by the participants, while BMI was calculated [body weight (kg)/height (m)^2^] based on assessments of height and body weight performed in the laboratory prior to participation in the experiment. The participant’s physical activity levels were assessed using an adjusted version of the leisure time exercise questionnaire (Godin and Shephard, 1985), which was modified in the caption to include transportation physical activity (i.e., walking or biking to reach different destinations). This adjusted version of the leisure time exercise questionnaire was used in a previous study and was found to correlate with objective assessments of physical activity by accelerometer (Calogiuri et al., 2013).

### Design and Procedure

The study was designed as a single-blinded experimental trial with two parallel groups. Participants were allocated to either one of the two experimental conditions with a 1:1 allocation ratio based on a predetermined sequence (i.e., the first participant was assigned to the LS IVE, the second to the HS IVE, the third to the LS IVE, and so on). This allocation procedure was chosen to ensure that an equal amount of experiments was carried out in each group in the same time period, as some participants were tested in June and some in August–October due to practical reasons. The allocation, which was determined by the first author, was stratified by sex to assure a balanced distribution of males and females in each group. This allocation was strictly based on the predetermined sequence, which was developed when the researchers were still unaware of the participants’ order, and never modified to accommodate participants’ preferences or characteristics. All participants were blinded to which condition they were allocated to and were unaware of the difference between the two IVEs. The experiment was performed in a controlled laboratory environment at Inland Norway University of Applied Sciences (Campus Elverum), with standardized temperature, ventilation, and lighting, and a high degree of sound insulation. The participants viewed the IVE while seated, and both videos lasted 10 min to approximate the exposure duration that give the largest effects on psychological outcomes according to the meta-analysis by Barton and Pretty (2010). No changes to methods were made after trial commencement.

The experimental procedures were performed as follows: (1) measurement of height and weight; (2) pre-exposure questionnaire, including physical activity affect scale and background information; (3) exposure to the IVEs, with continuous recording of HR from the third to the ninth minute of the IVE; and (4) post-exposure questionnaire, including SSQ, presence, perceived environmental restorativeness, physical activity affect scale, and enjoyment. In addition to the ones listed above, the study included additional measurements (i.e., a measure of future green exercise intention and assessments of postural stability during the first and last minute of video exposure), which are not presented in this paper.

### Analyses

The data were preliminarily explored in order to examine the frequency distribution of the various variables as well as identify possible missing values and outliers. The assumption of normality was evaluated by a Shapiro–Wilk test, which revealed that none of the outcome variables were normally distributed. Hence, a Mann–Whitney *U* test was used to investigate possible differences between the two IVE conditions for the SSQ scores, the eight items of presence, the four components of perceived environmental restorativeness, the four components of affect (expressed as delta values: post-exposure - pre-exposure), enjoyment, and HR. Potential between condition differences for the dichotomous measure of cyber sickness were analyzed by a chi-square test. In order to establish possible pre-to-post changes in the four different components of affect, a Wilcoxon signed-rank test was also applied to each of the affect components (Positive affect, Tranquility, Negative affect, and Fatigue). Lastly, a Spearman’s rank correlation coefficient (ρ) was used as an exploratory analysis to evaluate potential associations between cyber sickness, and presence, and all outcome variables. Because of the large number of between- and within-group comparisons, the level of significance was adjusted using the Benjamini–Hochberg procedure with a false discovery rate of 5%. Adjusted *p*-values are presented throughout the manuscript for between- and within-group comparisons. The correlation analysis was considered an exploratory analysis and, thus, the Benjamini–Hochberg procedure was not applied (Victor et al., 2010). All measurements were treated in accordance with standard procedures normally adopted for each instrument, and the overall statistical strategy was consistent with plans done prior to implementation of the study.

All outcome variables are presented as median (Mdn) and interquartile range (IQR). All statistical analyses were performed in IBM SPSS statistics 24 (IBM, New York, NY, United States). The significance level was set at *p* < 0.05.

## Results

The participants were generally considered active and healthy individuals with a mean (±standard deviation) age, height, weight, BMI, and modified leisure time exercise questionnaire score of 29.8 (±10.8) years, 173 (±10) cm, 76.1 (±14.9) kg, 25.2 (±3.3) kg/m^2^, and 57.2 ± 24.3 units, respectively.

**FIGURE 1:**
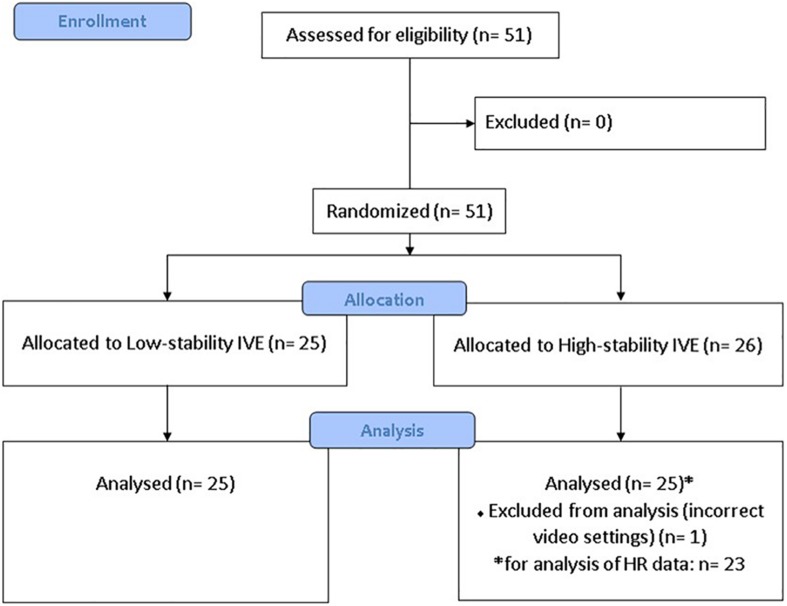
Flow diagram showing participants enrolled, allocated to condition, and included in the analyses (Schulz et al., 2010).

Three participants did not complete the full 10 min of video exposure due to high levels of cyber sickness. All three participants watched the LS video and discontinued after 3 min 56 s, 5 min 40 s, and 6 min 55 s. Data for all assessments were obtained for these participants and were included in the analysis (see flow diagram in Figure 1). Unfortunately, HR data were missing for two participants due to technical difficulties, and both of these participants were exposed to the HS condition.

### Cyber Sickness

**FIGURE 2:**
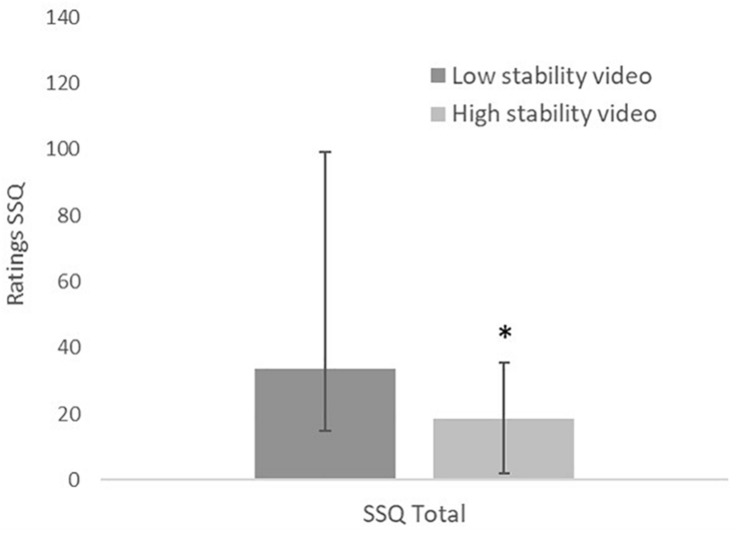
Comparison of the severity of cyber sickness symptoms measured with the Simulator sickness questionnaire after exposure to a low- or high-stability 360° video. SSQ Total = Total score (*n* = 50; medians and IQRs). ^∗^ = significant difference from low-stability video at the *p* < 0.05 level.

The dichotomous measure of cyber sickness revealed that 4 out of 25 participants (16%) in the HS condition and 12 out of 25 participants (46%) in the LS condition reported being sick. This effect was marginally not significantly different between the two conditions (χ^2^ = 5.88, adjusted *p* = 0.055). As shown in Figure 2, the Mann–Whitney *U* test on the total scores from the SSQ showed that participants in the HS condition reported significantly less severe symptoms of cyber sickness compared to participants in the LS condition (LS: Mdn = 33.66, IQR = 14.96–99.11; HS: Mdn = 18.70, IQR = 1.87–35.53, *U* = 179.0, adjusted *p* = 0.039).

Spearman’s rank correlation showed no significant correlations among SSQ scores and any of the components of presence or perceived environmental restorativeness. On the other hand, the analysis revealed medium to high correlations between SSQ scores and enjoyment and all components of affect (Table 3). No significant associations were found among the SSQ scores and HR.

**TABLE 3:** Spearman’s rho correlation between total cyber sickness score (top row) and the items of presence^1^, perceived environmental restorativeness^2^ (fascination, being away, coherence, compatibility), affect^3^ (positive affect, tranquility, negative affect, fatigue), enjoyment, HR, and background characteristics (*n* = 50).

	**Total SSQ**
Being there^1^	–0.14
Realism^1^	0.09
Sense of reality^1^	0.00
Awareness^1^	–0.04
Other people^1^	0.22
Noises^1^	0.19
Flatness^1^	0.02
Movement lag^1^	–0.15
Fascination^2^	–0.22
Being away^2^	–0.19
Coherence^2^	0.16
Compatibility^2^	–0.21
Positive affect^3^ (Δ)	–0.39^∗∗^
Tranquillity^3^ (Δ)	−0.35^∗^
Negative affect^3^ (Δ)	0.50^∗∗^
Fatigue^3^ (Δ)	0.63^∗∗^
Enjoyment	–0.48^∗∗^
HRmean	0.11
HRmax	0.14
Sex	0.17
Age	0.04
BMI	–0.15
Weekly PA	–0.11

*BMI = body mass index. Weekly PA = weekly physical activity. Δ = delta values. ^∗^Correlation is significant at the *p* < 0.05 level. ^∗∗^Correlation is significant at the *p* < 0.01 level.*

### Presence and Environmental Perceptions

**FIGURE 3:**
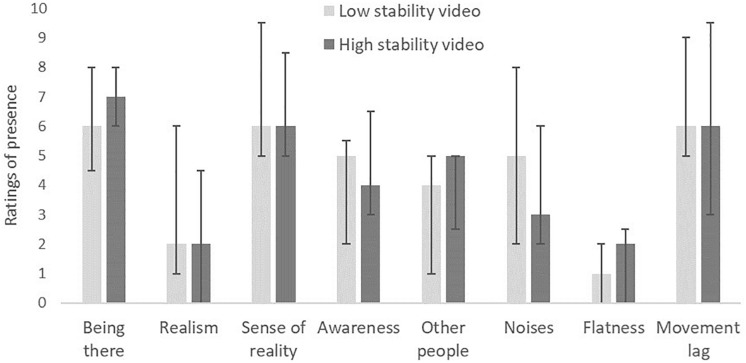
Comparison of ratings of presence associated with exposure to a low- or high-stability 360° video (*n* = 50; medians and IQRs).

**FIGURE 4:**
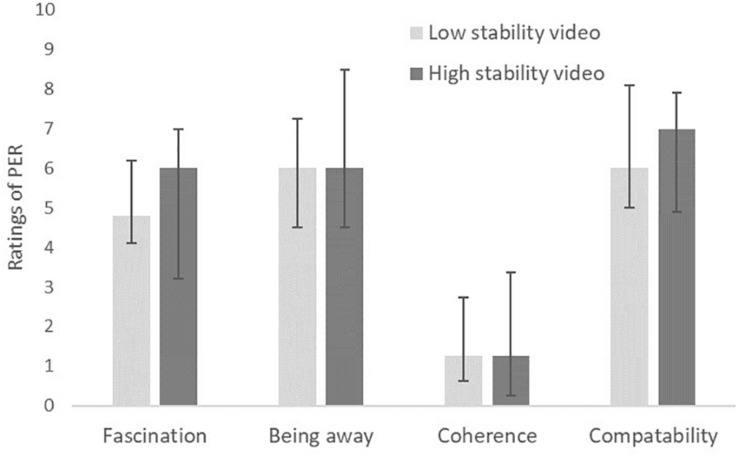
Comparison of ratings of the four components of the perceived restorativeness scale (PER) associated with a low- or high-stability 360° video (*n* = 50; medians and IQRs).

As shown in Figure 3, the sense of presence in the IVEs was similar in the two experimental conditions, with no significant between-condition differences for any of the eight items of presence (adjusted *p* = 0.179–0.899). Similarly, no significant differences between the two experimental conditions were found for the components of perceived environmental restorativeness (adjusted *p* = 0.589–0.938, Figure 4).

Spearman’s rank correlation revealed significant correlations between some items of presence and perceived environmental restorativeness, the four components of affect, and enjoyment (Table 4) including medium correlations between being there and three components of perceived environmental restorativeness and enjoyment, medium correlations between realism and one component of perceived environmental restorativeness and enjoyment, medium to high correlations between sense of reality and two components of perceived environmental restorativeness, a medium negative correlation between flatness and one component of perceived environmental restorativeness, and finally medium correlations between movement lag and the two negative components of the physical activity affect scale (Table 4). No significant correlations were found among the items of presence (Table 4).

**TABLE 4:** Spearman’s rho correlation between different components of presence (top row) and the components of perceived environmental restorativeness^1^, affect^2^, enjoyment, HR, and background characteristics (*n* = 50).

	**Being there**	**Realism**	**Sense of reality**	**Awareness**	**Other persons**	**Noises**	**Flatness**	**Movement lag**
Fascination^1^	0.40^∗∗^	0.34^∗^	0.26	0.13	0.04	–0.06	−0.31^∗^	–0.14
Being away^1^	0.38^∗∗^	0.26	0.46^∗∗^	–0.17	–0.09	–0.10	–0.17	0.10
Coherence^1^	–0.04	0.03	–0.04	0.19	–0.09	0.05	–0.05	0.23
Compatibility^1^	0.36^∗^	0.21	0.29^∗^	–0.01	0.00	–0.09	–0.18	0.06
Positive affect^2^ (Δ)	0.21	0.14	–0.10	–0.15	–0.03	–0.21	–0.12	–0.24
Tranquillity^2^ (Δ)	0.06	0.21	–0.12	–0.11	–0.19	–0.13	0.25	–0.13
Negative affect^2^ (Δ)	–0.07	–0.09	–0.06	0.12	0.07	0.04	–0.14	0.31^∗^
Fatigue^2^ (Δ)	–0.18	–0.16	–0.01	0.07	0.17	0.15	0.02	0.40^∗∗^
Enjoyment	0.28^∗^	0.30^∗^	0.06	0.17	–0.09	0.17	–0.18	–0.19
HRmean	–0.09	0.20	–0.09	0.10	0.07	–0.08	0.13	–0.20
HRmax	–0.09	0.24	–0.09	0.09	0.09	–0.10	0.08	–0.21

*Δ = delta values. ^∗^Correlation is significant at the *p* < 0.05 level. ^∗∗^Correlation is significant at the *p* < 0.01 level.*

### Psychophysiological Responses

The Wilcoxon signed-rank test showed that, after being exposed to the IVE, the participants in the LS condition had a significant deterioration of their affect state, with significant decrease in the ratings of Positive affect (Pre-exposure: Mdn = 3.67, IQR = 3.67–4.00; Post-exposure: Mdn = 3.33, IQR = 2.67–3.67; *Z* = −2.735, adjusted *p* = 0.002) and Tranquility (Pre-exposure: Mdn = 4.33, IQR = 4.00–4.67; Post-exposure: Mdn = 3.67, IQR = 2.67–4.33; *Z* = −2.972, adjusted *p* = 0.021) and a significant increase in the ratings of Negative affect (Pre-exposure: Mdn = 1.00, IQR = 1.00–1.33; Post-exposure: Mdn = 1.67, IQR = 1.00–3.00; *Z* = −3.194, adjusted *p* = 0.014). However, there was no significant change in ratings of Fatigue after the LS condition (Pre-exposure: Mdn = 1.67, IQR = 1.33–2.33; Post-exposure: Mdn = 2.00, IQR = 1.33–3.33; *Z* = -2.338, adjusted *p* = 0.063) as illustrated in Table 5. In contrast, no significant pre–post changes were observed in the HS condition for any of the components of affect (Positive affect, Pre-exposure: Mdn = 3.67, IQR = 3.00–4.00; Positive affect, Post-exposure: Mdn = 3.33, IQR = 3.00–4.00; Tranquility, Pre-exposure: Mdn = 4.33, IQR = 4.00–4.67; Tranquility, Post-exposure: Mdn = 4.00, IQR = 3.33–4.67; Negative affect, Pre-exposure: Mdn = 1.00, IQR = 1.00–1.67; Negative affect, Post-exposure: Mdn = 1.00, IQR = 1.00–1.67; Fatigue, Pre-exposure: Mdn = 2.00, IQR = 1.67–3.00; Fatigue, Post-exposure: Mdn = 1.67, IQR = 1.00–2.67; adjusted *p* > 0.05 for all comparisons). The Mann–Whitney *U* test of delta values for affect revealed significant differences between the two experimental conditions, with larger reductions of Positive affect (*U* = 174.5, adjusted *p* = 0.031) and larger increments of Negative affect (*U* = 146.5, adjusted *p* = 0.006) and Fatigue (*U* = 171.0, adjusted *p* = 0.028) after LS condition compared to HS. No significant difference between the two conditions was found for Tranquility (*U* = 226.0, adjusted *p* = 0.196). Similarly, the ratings of enjoyment after the HS condition (Mdn = 8.00, IQR = 6.00–9.00) were not significantly different compared to the LS condition (Mdn = 6.00, IQR = 5.00–7.00; *U* = 211.0, adjusted *p* = 0.136). No significant differences were found between the two experimental conditions for either average (LS: Mdn = 71.00, IQR = 64.00–77.00; HS: Mdn = 66.00, IQR = 62.00–77.00; *U* = 241.5, adjusted *p* = 0.551) or peak HR (LS: Mdn = 81.00, IQR = 72.00–91.00, *p* = 0.342; HS: Mdn = 76.00, IQR = 68.00–89.00; *U* = 252.0 adjusted *p* = 0.584).

**TABLE 5:** Pre–post changes of the four components of affect.

	**LS**		**HS**	
	**Mdn**	**IQR**	**Mdn**	**IQR**
**Positive affect**				
Pre	3.67	3.67–4.00	3.67	3.00–4.00
Post	3.33^∗∗^	2.67–3.67	3.33	3.00–4.00
**Tranquility**				
Pre	4.33	4.00–4.67	4.33	4.00–4.67
Post	3.67^∗^	2.67–4.33	4.00	3.33–4.67
**Negative affect**				
Pre	1.00	1.00–1.33	1.00	1.00–1.67
Post	1.67^∗^	1.00–3.00	1.00	1.00–1.67
**Fatigue**				
Pre	1.67	1.33–2.33	2.00	1.67–3.00
Post	2.00	1.33–3.33	1.67	1.00–2.67

*LS = low-stability video. HS = high-stability video. ^∗^ = significant difference from pre-values at the *p* < 0.05 level. ^∗∗^ = significant difference from pre-values at the *p* < 0.01 level.*

## Discussion

The results of the present study demonstrate that the 360° video characterized by high camera stability induced significantly lower levels of cyber sickness compared to the 360° video characterized by low camera stability, which is in support of our main hypothesis. The lower levels of cyber sickness in the HS condition were not accompanied by a significant difference in presence, which contradicts our secondary hypothesis 1.1. For our last hypothesis (1.2), which postulated that a reduction in cyber sickness should be accompanied by an improved psychophysiological response, the results are partly in favor of the hypothesis as we found a more positive psychological response in the HS condition, but the physiological measures were similar between the two conditions. These results were obtained in two 360° videos that were perceived as having similar environmental characteristics as indicated by the similar values reported between the two conditions for the components of perceived environmental restorativeness. Furthermore, our exploratory correlation analysis revealed several possible pathways that can help explain the complex relation linking cyber sickness, environmental perceptions, and psychophysiological responses.

The total SSQ score was substantially lower after the HS condition compared to LS, which suggests that improving camera stability and thereby reducing camera oscillations reduce severity of cyber sickness symptoms in 360° videos. These results support the findings of previous provocation studies examining the impact of severe levels of oscillations on cyber sickness using a head-mounted display (Lo and So, 2001; Bonato et al., 2009). Lo and So (2001) found that a severe increase in either pitch, yaw, or roll increased symptom severity with no differences between axis in a head-mounted display with a limited field of view (48° horizontal, 36° vertical). Bonato et al. (2009) confirmed that severe levels of oscillations induce cyber sickness, and further revealed that oscillations along two different axes simultaneously increased symptom severity compared to single-axis oscillation in a head-mounted display with a 60° field of view. Our results are in line with the findings of Lo and So (2001) and Bonato et al. (2009) for 360° videos and further demonstrate that also low levels of oscillations influence cyber sickness. This notion is in line with the Sensory Conflict theory proposed by Reason and Brand (1975). As outlined in the introduction, it is believed that oscillations causes a sense of self-motion in the opposite direction of the oscillation, which causes a conflict between visual inputs and vestibular and proprioceptive inputs. In the present study, the HS condition produced less camera oscillation along all three axes, which may have reduced sensory conflict. This potential reduction in sensory conflict may explain the lower symptom severity after the HS condition. In contrast to symptom severity, the occurrence of cyber sickness was not significantly different between low and high stability. However, the effect was borderline significant even after applying the Benjamini–Hochberg procedure (*p* = 0.055), which suggests that scene stability may influence occurrence of cyber sickness and that the issue is worthy of further investigation.

The lower symptom severity (total SSQ score) and potentially less sensory conflict suggest that the HS condition should have induced higher levels of presence compared to LS, as a study by Slater et al. (1995) suggests that reduced sensory conflict may improve presence (Slater et al., 1995) and a recent review found an inverse relationship between presence and cyber sickness (Weech et al., 2019). This was not the case and participants reported similar levels of presence regardless of which video they had viewed. Early research in VR proposed the logical argument that lower levels of sensory conflict would lead to higher levels of presence (Slater et al., 1995). This proposal is yet to be backed by rigorous scientific evidence, due to the challenge of directly measuring sensory conflict, but the idea has still carried on into more recent research (Weech et al., 2019). As stated above, we proposed the idea that the HS condition induced less sensory conflict compared to LS. These potentially lower levels of sensory conflict were not accompanied by higher levels of presence, which leads to one of two logical conclusions. Either lower levels of oscillations do not induce lower levels of sensory conflict or the idea that lower levels of sensory conflict increase presence is false. Unfortunately, the present study cannot make a definitive statement regarding this matter. However, our results definitively challenge the proposed relationship between cyber sickness and presence (Weech et al., 2019), as the between condition difference in cyber sickness was not accompanied by a difference in presence and there were no significant correlations between the two concepts. The lack of coherence with the conclusions by Weech et al. (2019) may have several explanations. Most importantly, Weech et al. (2019) acknowledges some limitations in the literature they reviewed: most of the literature had methodological limitations, such as limited sample size; most of the research identified supported an inverse relationship, but several papers also supported a positive relationship or no relationship; the review also identified several potential moderators of the relationship, such as sex, display factors, and context. In addition to the limitations in current research identified by Weech et al. (2019), it is also acknowledged that presence is a complex characteristic that is influenced by a multitude of factors, such as environmental interaction, synchrony of sensory stimuli, and fidelity of the virtual environment (Weech et al., 2019). Thus, it is possible that other aspects of the IVE, in addition to camera stability, must be improved in order to increase presence. Nevertheless, the present study does not support the proposed relationship between cyber sickness and presence and suggests that improved camera stability by itself does not result in higher levels of presence.

The fact that no differences were found between the HS and LS condition with respect to perceived environmental restorativeness is not surprising, as this measure is strongly dependent on the characteristics of the environments in which the 360° videos were filmed and should thus be interpreted as our efforts to reproduce highly comparable IVEs being successful. At the same time, this finding also suggests that, similarly to what was found for presence, the level of camera stabilization is unlikely to influence the viewers’ perceptions of the virtual environment. Furthermore, the finding of the present study not only shows the paramount impact of camera stabilization in avoiding negative affective responses in the viewers of an IVE but also confirms the central role of cyber sickness in explaining such responses. It has to be stressed, however, that even though the HS condition was associated with significantly more positive affective responses than the LS condition, different from what we would expect from an *in vivo* situation, it was yet unable to induce significant improvements in the participants affect profile. In a previous study, we found in fact that an actual walk of the same duration in the same (real) environment where the 360° was filmed induced improvements in the participants’ affect state, who reported a significant reduction of Fatigue and Negative affect after the walk as compared with before the walk (Calogiuri et al., 2018). This is in contrast with a previous study by Chirico and Gaggioli (2019), who found that the emotional responses to viewing a natural landscape *in vivo* or in form of IVE were not significantly different. In another study, Yu et al. (2018) compared participants’ responses to viewing a natural vs. an urban setting in IVE and found some similarities to what would be expected *in vivo* (e.g., a reduction of negative emotions in the natural IVE as opposed to an increase in fatigue in the urban IVE). However, unlike trials *in vivo*, no difference between the two IVEs was observed with respect to physiological measurements (blood pressure, salivary α amylase, and HR variability). In the present study, the lack of influence of improved camera stability on presence (which remained somewhat limited) may also contribute to explain the inability of the virtual walk in the HS condition to provide psychophysiological outcomes similar to those expected *in vivo*. Presence is considered a key element of a successful IVE (Steuer, 1992) and research within various fields have linked an IVE’s ability to induce feelings of presence to the ability to produce the desired effect of the specific IVE, e.g., within analgesia (Triberti et al., 2014) and treatment of anxiety and phobias (Ling et al., 2014; Botella et al., 2017). Since ratings of presence were similar in the HS and LS conditions, it was not surprising that the participants’ psychophysiological responses were similar as well. Remarkably, and in line with our previous study (Calogiuri et al., 2018), the ratings of enjoyment and all components of the physical activity affect scale were moderately to highly correlated with total SSQ score, which emphasize the paramount role played by cyber sickness in modulating the viewers’ affective responses to the IVE exposure. At the same time, significant medium correlations were also observed among enjoyment and two items of presence, namely, being there and realism. Based on the assumption that presence is a key element of an effective IVE, these findings suggest that the feeling of being there and realism were particularly important to the participants’ rating of enjoyment. Similarly, three components of perceived environmental restorativeness were moderately to highly correlated with several items of presence, which suggest that improved feelings of presence are closely associated with the restorative value of nature IVEs.

It should be noted that there were considerable individual differences in the response to the IVEs presented in this study, especially for SSQ. This phenomenon is clearly illustrated by the size of IQRs and by the fact that three participants were unable to complete the 10 min of exposure to the LS condition due to high levels of cyber sickness, while others could view the same video without experiencing any symptoms of cyber sickness. Other studies have also reported large inter-individual differences in the extent to which different individuals respond to virtual environments in terms of susceptibility to cyber sickness (Akiduki et al., 2003; Curtis et al., 2015; Gavgani et al., 2017). These individual differences are not fully understood, but research suggests that genetics (Hromatka et al., 2015), sex (Munafo et al., 2017), visual acuity (Allen et al., 2016), and postural control (Munafo et al., 2017) may explain some of the inter-individual variability. In the present study, cyber sickness was not associated with sex, or any of the participants’ background characteristics (age, BMI, and physical activity levels), which suggests that other factors caused the inter-individual variation. Further research that contributes to a better understanding of this phenomenon, and how it relates to the underlying causes of cyber sickness, is pivotal to shed more light on the issue of cyber sickness in relation to the application of IVE in green exercise research and practice, as well as and in the field of VR in general.

### Strengths and Limitations

The primary strengths of this study relate to important characteristics of its design, such as blinding of participants and rigorous experimental procedure, which allowed strict control of most confounding factors such as expectations, carryover effects, temperature, noises, and lighting. A possible limitation was the fact that that the content of the IVEs was not exactly identical, as the videos were recorded at different times. Although measures were taken to make the content as similar as possible (see section “IVE Technology and Experimental Conditions”), some minor differences such as light conditions, ambient noises, placement of objects, and activities of other people passing by were unavoidable. These differences were considered minor and the impact on the outcome of the present study should be minimal, as corroborated by equivalent ratings of perceived environmental restorativeness in the two conditions. The sample size in the present study was calculated *a priori* for our main outcome (total SSQ score) and the statistical power should be satisfactory for this measure, but the sample size may still have been too small to obtain adequate power for the other measures included in this study. Generalizability of the findings might be limited, as our participants were healthy adults, and it is uncertain whether the findings apply to VR users with pre-recorded clinical conditions (e.g., severe sight deficiencies, infections of the vestibular system, or other health problems). The possibility of occurrence of type I error should also be considered for the correlation analysis given the relatively large amount of statistical tests performed. However, this analysis was meant to reveal potential associations to be considered for further investigation.

## Conclusion

In the present study, we compared two different IVEs created by filming 360° videos with different stabilization techniques, leading to different amounts and frequency of small-magnitude oscillations. The findings show that a higher degree of scene stability in an IVE is paramount to reduce severity of cyber sickness symptoms, thus avoiding negative affective responses. Nevertheless, when comparing our findings to findings in studies of real nature, it is clear that even a highly stable IVE was ineffective in providing psychophysiological benefits equivalent to those expected *in vivo* (i.e., during a real nature walk). These findings not only demonstrate that technological advancements can improve the effectiveness of IVE in green exercise research and related areas but also show the complexity of the human–technology interaction and that more research and further technological advancement are needed before green exercise experiences can be sufficiently replicated in laboratory conditions.

## Data Availability Statement

The datasets generated for this study are available on request to the corresponding author.

## Ethics Statement

Ethical review and approval was not required for the study on human participants in accordance with the local legislation and institutional requirements. This study was registered in the Norwegian Centre for Research Data (reference number: 60451) and conducted in accordance with the Declaration of Helsinki. All patients/participants provided their written informed consent to participate in this study.

## Author Contributions

SL and GC contributed to the study concept, study design, statistical analysis, and revision of the manuscript. SL conducted the experiments and drafted the manuscript. Both authors approved the final version of the manuscript.

## Conflict of Interest

The authors declare that the research was conducted in the absence of any commercial or financial relationships that could be construed as a potential conflict of interest.
